# Use of evidence and expertise in UK climate governance: the case of the Cumbrian Coal Mine

**DOI:** 10.14324/111.444/ucloe.000068

**Published:** 2024-02-06

**Authors:** Rebecca Willis

**Affiliations:** 1 Lancaster Environment Centre, Lancaster University, Lancaster, UK

**Keywords:** climate, evidence, expertise, coal, steel, Climate Change Act, planning, Cumbria, UK

## Abstract

There is an overall scientific consensus that no new coal mines can be developed, if the Paris Agreement to limit global temperature rises is to be met. Yet in December 2022, following a lengthy Public Inquiry, the UK Government approved the development of Woodhouse Colliery in Cumbria. In doing so, it accepted the claim that the coal mine would be ‘zero carbon’ and could even result in lower global emissions overall. As this paper demonstrates, there is no independent evidence to support these claims, whilst a large body of independent evidence comes to the opposite conclusion. This paper uses the example of Woodhouse Colliery to examine the use of evidence and expertise in climate governance processes. It finds that the nature of expertise and evidence is not properly considered, and that there is ambiguity and confusion surrounding the implementation of the UK’s climate legislation, particularly the Climate Change Act. It also finds that the ways in which the decision-making process solicited and assessed evidence was flawed, promoting a ‘false balance’. This ambiguity and false balance provide scope for developers to argue the case for destructive developments, even while claiming adherence to climate ambitions. The paper concludes by suggesting reforms to governance processes, to provide a more transparent and credible implementation of policies to achieve the UK’s net zero target. Suggested reforms include clearer rules governing fossil fuel phase-out; greater transparency and better handling of conflicts of interest in decision-making; and devolution of climate responsibilities to local areas.

## Introduction

In 2022, eight years after it was first formally proposed, the UK government granted planning permission for Woodhouse Colliery, a proposed mine for metallurgical coal used in steelmaking. The route to approval (see Table 1) had been tortuous, with the mine approved on three separate occasions by the local authority, Cumbria County Council; a lengthy Public Inquiry; the launch of four legal challenges against the mine; and a great deal of media and political controversy. Much of the controversy has centred around the climate impacts of burning coal, the most carbon-polluting of all fossil fuels, in the UK – a country with comprehensive climate legislation, statutory targets to reach net zero greenhouse gas (GHG) emissions by 2050, and a strong commitment to the United Nations Framework Convention on Climate Change (UNFCCC) [1].

**Table 1.** Timeline of decision-making for Woodhouse Colliery

2014–2017	WCM develop plans and undertake consultation
May 2017	WCM submit application for detailed planning permission
March 2019	Cumbria County Council development control committee vote to approve the development
June 2019	UK Parliament legislates new target of net zero GHG emissions for the UK; legal challenge against WCM issued by Keep Cumbrian Coal in the Hole (KCCH)
October 2019	Cumbria County Council development control committee vote to approve the development
Nov 2019–Feb 2020	KCCH request a Judicial Review challenging the decision; this is granted
May 2020	KCCH withdraw their challenge as Cumbria County Council say they will reconsider the application
October 2020	Cumbria County Council development control committee vote to approve the development
December 2020	The Climate Change Committee (CCC) publish the Sixth Carbon Budget; Cumbria County Council say they will once again reconsider the proposal
March 2021	The Secretary of State ‘calls in’ the decision, i.e., states that it will be determined by the Government, following a Public Inquiry
September 2021	Public Inquiry takes place; two organisations play a formal role in opposing the mine: South Lakes Action on Climate Change (SLACC) and Friends of the Earth (FoE)
December 2022	Secretary of State issues planning permission for Woodhouse Colliery
January 2023	SLACC and FoE request a Statutory Review of the Secretary of State’s decision
May 2023	The request for a Statutory Review is turned down, but then granted on appeal. This Review will take place in the High Court; as of November 2023, a date has not been set

This paper reviews the decision-making process for Woodhouse Colliery, and assesses the lessons for climate governance, in the UK and more widely. I begin, in the section on The scientific consensus on climate change and fossil fuel extraction, with a summary of scientific evidence and international agreements on climate change, GHG emissions and fossil fuel extraction. In the section on UK climate governance: the state of play, I review the UK’s system of climate governance, centred around the 2008 Climate Change Act (CCA). In the section Woodhouse Colliery: climate claims and counter claims, I summarise the arguments put forward by West Cumbria Mining (WCM), in making the case that the mine would not adversely affect climate change; and state how these claims were countered. In the section How evidence was presented and used in the Public Inquiry, I then analyse some common threads in the way that evidence was presented and used in the Public Inquiry. Three tendencies are identified: first, imbalances in the status of expertise, in that, whereas WCM relied on commercial consultants, opponents of the mine were professionals with independent standing in academia or public life. Second, the exploitation of the ambiguity contained within UK climate legislation; and third, the tendency to ‘false balance’, giving equal weight to arguments for and against the mine, rather than assessing the state of evidence. The combination of these tendencies, it is argued, led to a decision in favour of the mine.

In the section Doubt and delay: strategies to question and limit climate action, the case of Woodhouse Colliery is placed in a global context and is shown to be part of a wider pattern of delay and ambiguity in climate action, in part orchestrated by powerful economic interests. In the Conclusions, the paper concludes with an assessment of changes needed to legislation and approaches to climate change, in the UK and more widely, if global climate goals are to be met.

As this paper is about the use of scientific and expert evidence in governance processes, it is important for myself, as the author, to be transparent about my own position. My expertise lies in the field of climate governance: the process by which societies and polities agree rules and strategies to combat climate change. The decision-making process around Woodhouse Colliery provides an example of this governance in action, and as such highlights many areas that could be improved, and indeed must be improved if the UK is to meet the targets it has enshrined in law.

I have been involved in the case directly, in two ways. I have provided media comment, based on the analysis that I set out in this paper. I have also assisted independent expert witnesses in providing evidence to the Public Inquiry, on areas including the link to climate legislation; prospects for steel industry decarbonisation; and international diplomacy issues. These experts have all spoken against the proposed development. This is set out in the section Woodhouse Colliery: climate claims and counter claims below. My involvement is based on my, and others’, assessment of the evidence. As an independent academic, my role is to assess evidence and give a clear account of its implications, as well as offering clarity about where uncertainties exist, or where there is limited evidence.

My media involvement, and my involvement in the Public Inquiry process, shows that I have a clear, publicly-stated position against the mine. This is based on my assessment of the evidence, which I set out in this paper. It is not my role to stay neutral unless such neutrality is justified by the evidence. If evidence on climate science and governance were different, and suggested that the mine could be justified, my account would reflect this. As I show in the section UK climate governance: the state of play, this is not the case.

I chose to publish this paper in a journal with an open peer-review process. This allows anyone to scrutinise the evidence I use, and the position I take. I actively sought comment from opponents to the mine and asked for evidence to substantiate their position. If there are errors of fact or judgement in the case I set out, I pledge to correct them transparently. I hope that this paper, and the peer-review process, will spark a useful debate about the role of evidence in climate governance.

## The scientific consensus on climate change and fossil fuel extraction

The 2015 Paris Agreement on Climate Change, signed by 195 parties including the UK, commits to stabilising the global climate to ‘to well below 2°C above pre-industrial levels and pursuing efforts to limit the temperature increase to 1.5°C’ [2], in order to limit dangerous climate change. The 2021 Glasgow Pact reaffirms this goal and develops more detailed plans for its achievement [3].

The implications of this global agreement for fossil fuel extraction are clear. The Intergovernmental Panel on Climate Change (IPPC) states that there is a linear relationship between GHG emissions and temperature rise, leading them to estimate in 2020 that only a further 500 gigatonnes of carbon dioxide (GtCO_2_) could be emitted, to have a 50% chance of limiting warming to 1.5°C [4]. This is the remaining ‘carbon budget’ that can be emitted if we are to have a fair chance of stabilising global temperatures. The total amount of emissions from developed reserves of oil, gas and coal, defined as ‘the cumulative quantity of oil, gas and coal that companies have already discovered and for which a financial and regulatory commitment to extraction has been made’, is estimated at 936 Gt CO_2_, almost double the remaining carbon budget for 1.5°C. Coal accounts for nearly half of this, at 446 Gt CO_2_ [5]. Thus, if the fossil fuels from developed reserves were extracted and burned, this would take us well over the global carbon budget. Existing developed reserves will need to remain unused if we are to keep to global temperature goals. Removing carbon dioxide from the atmosphere cannot happen at a scale significant enough to change this basic predicament [6]. The International Energy Agency estimates that only 0.004Gt CO_2_ is currently removed, predicted to rise to 1.6Gt CO_2_ by 2030 and 7.6Gt CO_2_ a year by 2050 [7].

Any new sites of fossil fuel extraction would only add to this problem. A range of studies and reports have concluded, therefore, that new fossil fuel extraction sites are incompatible with the Paris Agreement, although the Agreement itself does not explicitly prohibit such sites. Reports by the United National Environment Programme [8]; the International Energy Agency [7]; as well as academic studies [9,10] show that no new extraction facilities such as oil or gas wells, or coal mines, can open, if we are to stay within the globally agreed carbon budget; and existing sites will have to reduce their production. This is a matter of arithmetic, not opinion. In the words of UN Secretary General Antonio Guterres, ‘climate activists are sometimes depicted as dangerous radicals. But the truly dangerous radicals are the countries that are increasing production of dangerous fossil fuels. Investing in new fossil fuel infrastructure is moral and economic madness’ [11].

## UK climate governance: the state of play

The UK was the first country to set statutory (legally binding) targets to guide GHG reduction at a national level. The CCA, passed in 2008, initially set a target of 80% GHG reduction in GHGs, by 2050, from a 1990 baseline. Under the Act, Parliament must agree five-yearly ‘carbon budgets’, essentially interim targets to ensure progress toward the 2050 target. In setting carbon budgets and developing strategies to meet them, Government and Parliament are advised by the independent advisers, the Climate Change Committee, also established under the 2008 Act. In 2019, the Act was amended, setting a more stringent goal of ‘net zero’ GHG emissions by 2050, with ‘net zero’ meaning that any emissions of GHGs must be matched by equivalent levels of GHG removals, through changes to land use such as increased tree planting, and through mechanical removal, such as carbon capture and storage (CCS).

While the CCA is a comprehensive piece of legislation, setting economy-wide targets, it has a number of significant weaknesses and ambiguities. These include: (1) a lack of clarity over the contribution of different sectors of the economy to GHG reduction; (2) ambiguous and unclear links between the CCA and planning policies; (3) statutory targets are set at a national level only, with ambiguity over the expected contribution of local administrations; (4) in terms of GHG accounting, the targets relate to GHG emissions from within UK territorial borders, not emissions in other jurisdictions which could reasonably be seen to be resulting from UK-based activities; and (5) there is no clarity over the role or extent of GHG removals in achieving the 2050 target. These weaknesses and ambiguities, which are detailed below, are all illustrated in the example of Woodhouse Colliery, as discussed in the sections Woodhouse Colliery: climate claims and counter claims and How evidence was presented and used in the Public Inquiry below.

### Contribution of different sectors of the economy to GHG reduction

The targets for emissions reduction in the CCA are not broken down by sector of the economy, or by government department. One department, currently the Department for Energy Security and Net Zero, has overall responsibility for leading the UK’s climate strategy and meeting the targets. Achieving these targets requires action by other departments as well, yet there is no set process for managing decarbonisation across different departments and sectors [12]. The Climate Change Committee assesses evidence and provides advice on the role of different sectors of the economy, in effect offering targets for different sectors. For example, the sector pathway for steel implies that by 2039, unabated coal (burning coal without capturing carbon) must end, as described by Professor John Barrett in his evidence to the Public Inquiry (Climate Change Committee [13]; also see Woodhouse Colliery: climate claims and counter claims below). However, these sector pathways are merely advisory. The Climate Change Committee has identified the lack of clarity and responsibility, a ‘governance gap’, as a major risk to delivery of the UK’s net zero target. In their report on the Sixth Carbon Budget they state that there is a lack of clear roles and responsibilities for other departments, and for regulators, devolved and local government [13].

This ‘governance gap’ means that the contribution of different sectors of the economy to GHG reduction is not clearly delineated. The Climate Change Committee recently judged that there are credible plans in place for only 39% of the emissions reductions needed to meet the sixth Carbon Budget, with significant gaps or uncertainties in crucial areas including transport, industrial decarbonisation and land use [13]. This uncertainty directly affects the decision over Woodhouse Colliery, because it is not clear who should take responsibility for the GHG emissions from planning decisions (overseen by the Department for Levelling Up, Housing and Communities) or from the coal or steel industry (overseen by the Department for Business and Trade).

### The role of the planning system in relation to climate targets

Developments in England are governed by the National Planning Policy Framework (NPPF) (Ministry of Housing Communities & Local Government [14], revised 2021). The NPPF sets out what the Government’s planning policies are, and how they should be applied. This provides a framework within which local areas develop their own, locally-specific plans. In the case of Woodhouse Colliery, the relevant local plan was the Cumbria Minerals and Waste Local Plan. The NPPF states that ‘the planning system should support the transition to a low carbon future’ [14, p. 45]. However, there are ambiguities about how this ambition should be realised, and in particular, about whether ‘end use’ emissions (i.e., in this case, emissions from burning the coal mined in Cumbria) should be considered as part of the planning process. As a result, this issue has been argued through numerous legal cases, including over Woodhouse Colliery.

The NPPF also contains a presumption against coal extraction, stating that planning permission should not be granted for the extraction of coal, unless the proposal is ‘environmentally acceptable’, or if it provides ‘benefits which clearly outweigh its likely impacts’ [14, paragraph 217, p. 62]. However, the NPPF does not state how ‘environmentally acceptable’ should be defined or tested, or how to weigh up the benefits against likely impacts. As a result, again, these questions have been argued through numerous legal cases.

The decision on Woodhouse Colliery was taken through the planning system, ultimately through a Public Inquiry led by a Planning Inspector. The Inspector’s task was to rule on whether the proposal was lawful, under England’s current planning laws. The wider question, of whether the proposal is compatible with UK climate legislation or international climate agreements, was not considered directly, but only indirectly, that is, the extent to which planning policy reflects and implements climate legislation and agreements. Of course, developments must comply not just with planning law, but with all law. However, there is no clarity on the link between planning law and UK climate legislation, and the resulting ambiguity is deeply problematic for individual planning decisions, as examined in Woodhouse Colliery: climate claims and counter claims below.

### Local contributions to GHG reduction

UK local government currently has no specific statutory responsibility for GHG reduction. Responsibility for meeting the statutory net zero target (and interim carbon budgets) of the Climate Change Act lies with the national parliament and government, as well as the devolved nations (Scotland, Wales and Northern Ireland). Implicitly, it is clear from the Act that all local authorities – indeed, all branches of government – must play their part in meeting the overall target, but there are no clear roles, responsibilities or targets assigned to local authorities. Nevertheless, many local areas have set their own targets and plans. For example, Manchester has a target ‘to become a zero carbon city’ by 2038 [15]; London by 2030 [16]; and Cumbria by 2037 (note that in April 2023, following local government reorganisation, Cumbria County Council was split into two different authorities: Cumberland Council, and Westmorland and Furness Council) [17]. These local targets are not enshrined in law, and local authorities all measure and manage their climate impacts in different ways. This contributes to the overall complexity of achieving the UK’s climate goals. For example, it is unclear whether or how Cumbria’s target of net zero emissions by 2037 was taken into consideration in the planning decision for Woodhouse Colliery.

### Accounting for GHG emissions

In line with international conventions in GHG accounting, the statutory targets enshrined in the CCA relate to so-called ‘production’ emissions. GHGs are counted where the gases are actually produced and enter the atmosphere – these are ‘production’ emissions. It is also possible to account for GHGs at the point of consumption of goods. For example, the GHG emissions associated with manufacturing a laptop in China, but sold in the UK, are conventionally ascribed to China, as the place of manufacture. Yet to the extent that demand for such goods is driven by consumption patterns in the UK, the UK could be said to hold some responsibility for these emissions. The UK does acknowledge this, in that it publishes accounts of consumption-based emissions [18], but the Climate Change Act accounts for production emissions only. Another way in which GHGs could be measured is through so-called ‘extraction’ emissions: the point at which fossil fuels are extracted from the ground. Under international conventions, countries that extract coal, oil and gas for export do not account for the emissions that arise when the fuels are burned in a different country.

As an example, the emissions resulting from steel used in construction could be accounted for in at least three different places, and quite possibly in three different countries: the mine where the coal was extracted for steelmaking (extraction emissions); the steelworks that burned the coal to make steel (production emissions); or the building site where the steel is used in construction (consumption emissions). Under UNFCCC guidelines, only the production emissions from the steelworks count toward a country’s GHG inventory [19].

As with all accounting, conventions are necessary, to avoid double- or triple-counting of emissions. However, there is a danger that this hinders potential routes to GHG reduction. If extraction emissions were considered, and discouraged – through a carbon price, for example – this could influence steel manufacturers to look at alternatives such as hydrogen-based production methods. If consumption emissions were considered, this could influence the construction industry to source recycled steel or use less steel.

An over-reliance on production-based emissions accounting therefore risks discounting a number of feasible GHG reduction routes. It places an artificial boundary around an activity, such as coal mining, or the import of consumer goods, meaning that emissions from those activities can be ignored, even if there are steps that could have been taken to reduce emissions. In an acknowledgement of this, some countries and local areas have instigated particular policies and laws focussed directly on limiting extraction of fossil fuels, including France, US states and Wales [20].

### The role of GHG removals

The emergence of the concept of ‘net zero’ emissions has put the spotlight on the ‘net’ in net zero – in other words, the use of GHG removal technologies to compensate for GHG emissions. GHG removal options involve capturing and storing GHGs, either using ‘natural’ processes such as land-use changes – tree planting and soil management, for example – or ‘engineered’ processes, such as capturing and storing carbon dioxide from industrial processes. Nearly all scenarios outlining credible paths to net zero, including those developed by the International Energy Agency, the Intergovernmental Panel on Climate Change, and the UK’s Climate Change Committee, include a certain level of GHG removal [7,13,21].

There is a strong consensus that the total technical and economic potential for GHG removal is limited, and therefore it cannot be a substitute for GHG reduction. For the UK, the Climate Change Committee’s advice is that GHG removal should be used to compensate for so-called ‘residual emissions’ that are very difficult to eliminate, particularly from land use, agriculture and aviation (Climate Change Committee [13]; see also Anderson and Peters [6]).

In summary, the role played by GHG removals is limited, and should be seen as an addition to, rather than an alternative to, reductions in GHG emissions. However, the very conception of ‘net zero’ subsumes GHG removals and reductions in GHG emissions into one single metric, with the sense that one can be traded off against another [22]. This is the logic behind so-called ‘offsetting’ schemes offered to individuals and companies to ‘compensate’ for GHG emissions from aviation or buying vehicle fuel, for example. There is evidence that this approach to GHG removal actually hinders or discourages reductions in GHG emissions [23]. There is a strong case for separating out targets for GHG removals from reductions in GHG emissions to ensure that GHG removals are additional, not an alternative approach [22]. In the UK, this could be done through specifying targets for each, as part of the CCA budget-setting process. However, at present, there is no such clarity.

## Woodhouse colliery: climate claims and counterclaims

It is clear from basic scientific evidence (see The scientific consensus on climate change and fossil fuel extraction) that any new fossil fuel developments would result in emissions increases incompatible with the goals of the Paris Agreement. The UK is a signatory to the Paris Agreement, yet its government approved Woodhouse Colliery. How can this have happened? This section surveys the main claims, and evidence, put before the Public Inquiry into the coal mine, held in September 2021.

The Public Inquiry is explicitly tied to the planning system. The role of the Planning Inspector, who conducted the Inquiry, was to assess the development against planning legislation and guidance. Thus, it would not be enough to say, as demonstrated in The scientific consensus on climate change and fossil fuel extraction above, that the mine is incompatible with the UK’s climate commitments. Instead, the case must be made with reference to the complex relationship between planning law and climate commitments.

In presenting its case, WCM never stated opposition to the Climate Change Act, or the Paris Agreement. Instead, it made the case that the development was compatible with the UK’s responsibilities on climate [24]. This can be seen as an argument in three stages. First, they sought to show that the proposed development was permissible within planning law and guidance, as set out in the NPPF (see The role of the planning system in relation to climate targets above). Second, they implied that, because it was (as they claimed) permissible within planning law, logically it must be compatible with UK climate legislation more generally, including the Climate Change Act. Third, they claimed that because it was permissible within planning law, and that this implied it must be compatible with UK climate legislation, it must therefore follow that it has a neutral, or even positive, effect on climate change.

This argument would make sense if there were specified, transparent and undisputed links between planning legislation, climate legislation and overall climate impacts – in other words, if the ambiguities in legislation were minimal. However, as described in the section UK climate governance: the state of play above, this is not the case. The links between the Climate Change Act and the NPPF are disputed; there are also ambiguities about how GHG emissions should be accounted for.

Despite this situation, WCM’s arguments were largely accepted by the Secretary of State, Michael Gove, who stated in his decision letter approving the mine that the proposed development ‘would to some extent support the transition to a low carbon future’ and ‘would have an overall neutral effect on climate change and is thus consistent with Government policies for meeting the challenge of climate change’ [25, paragraph 38].

For the Secretary of State’s conclusion to be correct, all of the following claims put forward by the mine must be correct:

- WCM can only be held responsible for emissions from the mine site, not from emissions from burning coal;
- the mine will result in reduced transportation of coal, and lower greenhouse gas emissions due to more efficient facilities;
- coal will still be needed to make steel, and coal burning will be offset either through offsetting schemes or through emissions reductions elsewhere in the economy;
- offset schemes can be used to compensate for any residual emissions;
- coal from Cumbria will substitute for coal mined elsewhere, with other mines reducing production in line with increases from the new mine;
- consenting a coal mine will have no effect on international diplomacy or other countries’ commitment to climate action.

These claims, and the responses to them from those opposing the scheme, are described below. Each was the subject of lengthy documentation, and considerable debate during the Public Inquiry. As I discuss in the section How evidence was presented and used in the Public Inquiry, if UK climate legislation were clearer, these complex claims and counterclaims would not have needed to be played out in the Inquiry. For instance, the role of GHG removals (see section The role of GHG removals above) would not need to be discussed at length if the principles were set out explicitly in climate legislation. The lack of clarity created what I describe (see section False balance) as ‘false balance’, in which complex arguments for and against the mine, and claims about compatibility with ambiguous legislation, distracted from the fundamental point that further coal extraction is incompatible with aims of the Paris Agreement.

In describing the claims and counterclaims set out in the Public Inquiry, my aim is not to set out the issues in full, but to present an indication of the issues that were considered as part of the decision-making process. I only examine arguments relating to climate issues in this paper. The Public Inquiry also covered other issues, such as the future of the steel industry; employment considerations; other environmental issues; and other land use planning matters. These issues are undoubtedly important. However, if the mine contravenes the UK’s climate commitments, in the form of the Climate Change Act and the goals of the Paris Agreement, then logically it cannot go ahead. A breach of law cannot be justified through an appeal to other benefits.

### Only emissions from the mine site should be considered

In its Statement of Case, WCM states that ‘it is not appropriate to have regard to GHG emissions caused by the end use of the coal extracted from the proposed development at other facilities’ [24, p. 40]. In other words, WCM state that they should not be responsible for the emissions caused by burning the coal and should only have responsibility for the emissions from the mine site itself. As discussed (see section Local contributions to GHG reduction), this claim is based on the convention that GHGs are counted where they are emitted into the atmosphere, that is, where the coal is burned, not where it is extracted.

Respondents, including Professors Michael Grubb and John Barrett, disputed this, stating that these end-use emissions were a material consideration, given the need to take account of UK climate legislation in planning policy. The question of how end-use emissions should be taken into account in planning law is also the subject of a separate legal dispute, the ‘Finch’ case, which, as of November 2023, is being considered by the Supreme Court [26].

### Fewer imports; efficient facilities

Second, WCM’s Statement of Case says that ‘the proposed development will help support the transition to a low carbon future […] by removing reliance upon imported coking coal with a higher carbon footprint’ [24, p. 40]. Specifically, it states that the development will ‘reduce transportation emissions’ and ‘provide the opportunity to create a state-of-the-art mining facility with lower GHG emissions than other existing mining operations’ [24, p. 41].

These claims were disputed by respondents, including Professor Michael Grubb, Professor John Barrett and Professor Paul Ekins. They stated that the emissions from the mine site, and from coal transportation, were a tiny fraction of the emissions from burning the coal. There was also conflicting evidence about whether the coal would be used within the UK (thereby reducing imports) or whether it would be shipped elsewhere. Aspects of the mine’s own operations were critiqued, particularly the issue of methane emissions from the mine site.

### Coal will still be needed to make steel, with CCS

Third, WCM states that ‘coking coal is likely to continue to form part of a net zero compliant option for steel production’ [24, p. 41]. This was disputed by Professor Lars Nilsson, Professor Paul Ekins and Professor Stuart Haszeldine, who stated that steel companies were increasingly using hydrogen-based steelmaking, which did not require coal; and that more steel could be recycled using electric arc furnaces.

### Use of offsetting

WCM states that ‘where it is not possible to remove operational GHG emissions entirely, WCM will commit to ensuring that these residual emissions are offset’ [24, p. 41]. As described in the section The role of GHG removal above, it is not credible to claim that GHG removals can be used to ‘offset’ GHG emissions that could be otherwise reduced or avoided. WCM stated that it would use Gold Standard certified credits; however, the Gold Standard Foundation, which oversees the use of these credits, provided a letter to the Public Inquiry stating that it is ‘strongly against the further extraction of fossil fuels’ and that new coal mines are to be avoided [27].

### Coal will substitute for coal mined elsewhere

The WCM Statement of Case states that, although the end-use emissions (i.e., from burning the coal) should not be taken into account, even if they are taken into account, ‘there is a strong economic case for substitution’, that is, that Cumbrian coal would substitute for coal mined elsewhere. In other words, every tonne of coal extracted in Cumbria would result in a tonne of coal **not** being extracted elsewhere, thus not increasing the total amount of coal burned or GHGs emitted. WCM’s argument was supplemented by a report from consultants Ecolyse.

Professor Michael Grubb and other respondents disputed this case. Professor Grubb stated that it was highly unlikely that the opening of the Cumbria mine would result in reduced production in other mines, thus disputing the ‘substitution’ argument. He calculated that even if just 1% of the coal mined in Cumbria was additional, this would more than double the total emissions of the mine as estimated in the Ecolyse report. Similar arguments were put forward by Professor Paul Ekins, who presented peer-reviewed research on the price elasticity of coal, stating that WCM coal would decrease prices for metallurgical coal and therefore increase demand.

### Impact on international diplomacy

The WCM Statement of Case makes no mention of an argument used by opponents of the mine, that the UK’s permitting of the mine would send unhelpful political and diplomatic signals, making other countries less ambitious on climate. This argument was put forward by opponents to the mine, including Professor Grubb; Professor Sir Robert Watson; Lord Deben, Chair of the Climate Change Committee; and John Ashton, former UK Government Special Representative for Climate Change.

## How evidence was presented and used in the Public Inquiry

In this section, I draw out some patterns in the way that evidence was presented and used in the Public Inquiry, namely the status of expertise; the exploitation of ambiguity; and the creation of ‘false balance’.

### The status of expertise

As can be seen from Table 2, there was a notable imbalance in expertise on climate issues at the Public Inquiry. WCM relied on commercial consultants that they themselves had commissioned, including reports by consultancies Ecolyse and AECOM, and appearances in front of the Inquiry by Ms Caroline Leatherdale, a consultant focussing on environmental impact assessments, and Mr William Tonks, a mining ventilation specialist. By comparison, many of those expressing opposition to the mine had climate specialisms – these included Professor Michael Grubb, Professor Paul Ekins, Professor Sir Robert Watson, Professor John Barrett, John Ashton CBE and Lord Deben (see Table 2 for affiliations) and spoke in an independent capacity, not as paid consultants, using evidence from peer-reviewed or independent sources.

**Table 2.** Witnesses on the issue of climate change called before the Public Inquiry

Witnesses appearing for WCM	Witnesses appearing for South Lakes Action on Climate Change and Friends of the Earth UK
Ms Caroline Leatherdale, environmental adviser employed by WCM Mr William Tonks, specialist in mine ventilation, director of Bill Tonks Ventilation Services Ltd	Professor Sir Robert Watson, former Chair of the Intergovernmental Panel on Climate Change, former Chief Scientific Adviser to the Department for Environment, Food & Rural Affairs, former Chief Scientific Adviser to the World Bank, former Associate Director for Environment in the Clinton White House Professor Paul Ekins, professor of resources and environmental policy at the UCL Institute for Sustainable Resources, former adviser to the UK Parliament and the Climate Change Committee Professor Michael Grubb, professor of Energy & Climate Change at UCL, former member of the Climate Change Committee, former adviser to the UK Office of Gas and Electricity Markets Professor John Barrett, Professor of Energy & Climate Policy, University of Leeds; adviser to the UK Department for Business, Energy & Industrial Strategy; lead author for the Intergovernmental Panel on Climate Change working group III ‘mitigation of climate change’

An assessment of both written and verbal evidence heard during the Public Inquiry thus suggests that the weight of evidence strongly supported the position that the climate impacts of the mine are negative, and indeed contrary to the UK’s climate objectives. This ‘weight of evidence’ can be judged by levels of expertise of witnesses; quality of evidence as judged by use of peer-reviewed data, for example, and independence, that is, professionals with independent standing in academia or public service, who had not been commissioned or paid as consultants to give evidence.

This is not to question the expertise or integrity of the consultants employed by WCM. I am not claiming that the consultants purposefully misled the Inspector, but that, by the nature of their commission, they provided specific, limited answers to the specific, limited questions they were given. Preparing a consultancy report in response to a specific brief is a different process to preparing an independent statement based on peer-reviewed evidence.

### Exploiting legislative ambiguity

As set out in the section UK climate governance: the state of play above, there are clear limitations and ambiguities contained within UK climate legislation, as well as within the planning system. These limitations and ambiguities allow developers to claim that their projects are allowable under the legislation. With reference to each of the weaknesses and ambiguities described in that section:

- Ambiguities surrounding **the contribution of different sectors of the economy** (see section Contribution of different sectors of the economy to GHG reduction above) provides room for WCM to claim that the emissions from their development should be allowed, with the required national GHG reductions coming from unspecified actions elsewhere.
- The ambiguities in **the planning system** (see section The role of the planning system in relation to climate targets above) and specifically the NPPF, create confusion about whether the full climate impacts of any given development should be considered in a specific planning decision.
- As there is no clear legislation or policy on **local contributions to GHG reduction** (see section Local contributions to GHG reduction above), Cumbria County Council is not required to account for the emissions from the mine in its own climate strategy.
- In terms of **accounting for GHG emissions** (see section Accounting for GHG emissions above), the lack of targets or policy covering extraction of fossil fuels allows WCM to claim that they should only shoulder responsibility from the mine site itself, not from the end use of the coal.
- In terms of **greenhouse gas removals** (see section The role of GHG removals above), the lack of clarity on the contribution of removals to the overall target allows WCM to make the claim that its emissions can be ‘offset’ through removals.

These arguments can be seen throughout WCM’s documents and argumentation in the Public Inquiry. In summary, WCM say that ‘the overall responsibility for the economy-wide transition to a low carbon society and the policies that are required to support that transition is the responsibility of the UK Government’, and that ‘these matters must be considered holistically, rather than on a case-by-case basis, through the determination of planning applications’ [24, p. 29]. Where there is so much ambiguity and complexity, it becomes possible to claim that one specific development cannot be held to account.

### False balance

In making its central claim that the climate impact of Woodhouse Colliery is neutral, WCM’s strategy can be seen as promoting so-called ‘false balance’. False balance can be defined as ‘presenting two sides of a debate as more equal than is justified by the evidence’ [28, p. 64]. False balance has been much discussed in regard to media coverage of climate science, when media outlets give equal airtime to scientists supporting and opposing the scientific consensus on climate change, despite the presence of an overwhelming consensus overall [29,30]. Thus, in a debate about climate impacts, a climate scientist representing the consensus position is paired with someone who does not accept this consensus, even though this position is at odds with the weight of scientific evidence. False balance sometimes comes about because media producers believe that it is important to represent ‘both sides’ of a debate; it may also come about because of a particular agenda that the media outlet is pursuing.

The use of false balance in the legal case over Woodhouse Colliery is similar. In the case, mine supporters made claims about the supposedly ‘positive’ climate impacts, opening up a debate between two opposing views, even when this debate is not justified by the weight or quality of evidence. Instances of false balance include, first, the statement that offset schemes can be used to ‘compensate’ for any residual emissions, when there is a clear scientific consensus that this is an inappropriate use of GHG removals (see sections The scientific consensus on climate change and fossil fuel extraction, The role of GHG removals and Use of offsetting above). Second, the statement that the mine would result in GHG savings because of reduced transport costs, and because coal from Cumbria will substitute for coal mined elsewhere, was not substantiated by evidence (see section Fewer imports; efficient facilities above). Lastly, the idea promoted by WCM that the coal mine would be a ‘zero carbon coal mine’ is not supported by convincing evidence, and relies on offsetting, which, as described above, is discredited.

These statements, even if badly served by underlying evidence, must be considered and debated. Each must be examined and rebutted. In the media coverage on the coal mine, these claims were, indeed, discussed at length. Debates often involved two contributors, one speaking in favour of the mine, and one against.

Added together, this contributes to an overall false balance – the assertion that there is a debate to be had about whether a new coal mine can be opened. Thus, the simple evidence set out in the section The scientific consensus on climate change and fossil fuel extraction, that any new coal mine is not compatible with the aims of the Paris Agreement, is replaced by a complex series of claims which, even if not supported by the evidence, serve to provide the impression that there are two, evenly-balanced ‘sides’ to the debate.

## Doubt and delay: strategies to question and limit climate action

In the section Woodhouse Colliery: climate claims and counterclaims, I set out the way in which WCM could put forward their argument that this mine has an overall positive effect on climate change, despite overwhelming evidence to the contrary. I now place this case in a wider context of the strategies employed by high-carbon economic interests, to make a case for continued exploitation of fossil fuels.

There is a well-documented history of companies involved in fossil fuel extraction opposing the scientific consensus on climate change, through funding and cultivating links with think-tanks, policy institutes and commentators who oppose the consensus [31]. The strategy, for many years, was to raise questions and promote debate about the science, thereby obscuring the clear scientific consensus on anthropogenic global warming. These tactics had been learned from the tobacco industry, who had, for many years, sought to promote doubt about the links between smoking and serious harm to health.

The strategy worked. The Intergovernmental Panel on Climate Change published its first report documenting the scientific consensus on climate change in 1990. It took nearly 30 years for the BBC to tell its editors that it was not necessary to include outright deniers of climate science in order to achieve ‘balance’ [32]. In the intervening decades, the ‘false balance’ arguments about whether climate change was happening or not, squeezed out the very necessary debates of how to respond to climate change and reduce GHG emissions.

More recently, the science of climate change has largely been accepted, even by companies involved in fossil fuel extraction (it is, however, worth noting that doubt about climate science still has a strong foothold in media and politics, particularly in the US, where many Republican politicians openly express doubts [33,34]). Tactics have shifted from denying the science outright, to opening up a range of often spurious debates about what the responses should be. This new approach has been dubbed ‘Discourses of Delay’ [35]. Such discourses include shifting responsibility for action – ‘emissions reductions can come from elsewhere’; comparisons – ‘our carbon footprint is trivial compared to others’; technological optimism, including a faith in GHG removals; and ‘fossil fuel solutionism’ in which fossil fuels are seen as a bridge to a zero-carbon future. It is important to note that these arguments are not always entirely wrong or used intentionally to slow climate action. As Lamb et al. make clear, ‘discourses of delay often contain partial truths and may be put forward in good faith’ [35, p. 2–3]. However, ‘in the absence of high-quality public deliberation, and in the hands of interest groups fighting against regulation, our concern is that discourses of delay will disorientate and discourage ambitious climate action’ [35, p. 3].

This is exactly the approach taken by WCM, and the mine’s supporters more generally. WCM did not question the science of climate change, nor the UK’s specific net zero target, the Climate Change Act, or its international obligations under the Paris Agreement. Instead, their approach was to say that they agreed with the need for climate action, but that their own project was legal, and would not have a negative effect. A whole set of complex arguments (summarised in the section Woodhouse Colliery: climate claims and counterclaims) were deployed, introducing complexity and confusion. When combined with the ambiguities of UK climate legislation (see section UK climate governance: the state of play), this meant that the mine’s opponents had to engage in detailed debate about each of these arguments – a much more difficult and complex job than simply stating that the mine is incompatible with the aims of the Paris Agreement (see section The scientific consensus on climate change and fossil fuel extraction). Overall, as set out in the section False balance above, this contributes to a false balance – the idea that there is any debate to be had over whether a new coal mine should go ahead.

Having been closely involved in the mine debate over several years, I saw this pattern of complexity, doubt, delay and false balance – enabled by the ambiguities and inconsistencies of UK climate legislation – play out many times over, in the protracted legal process and in media debates. When asked for media comment on the mine, I tried to put forward two points: first, that the mine was incompatible with the aims of the Paris Agreement; and second, highlighting the tactics of doubt and delay used by mine supporters. However, the questions I was asked were never about these general points, but about the detail of specific issues – complexity instead of simplicity.

## Conclusion

This paper set out to answer the question of how a coal mine could be consented in a country with world-leading climate legislation, in the face of clear evidence that the opening of further fossil fuel extraction sites is not compatible with the aims of the Paris Agreement, and at a time of rapidly worsening climate impacts. It found that the case for the mine was made through exploiting ambiguities in the UK’s climate legislation, in particular the unclear links between planning policy and the Climate Change Act; and through the introduction of complex, under-evidenced arguments which combined to create a false balance – the impression that there is a debate to be had about whether or not the mine contravenes climate ambitions.

As argued in the section How evidence was presented and used in the Public Inquiry, the case of Woodhouse Colliery is an example of a wider tendency to foster complexity, doubt and delay in climate decision-making. As such, it should not be seen as a one-off aberration, but an indication of a deeper problem. Similar arguments are being played out in other domains. These include arguments for opening new oil and gas extraction sites in the North Sea, which are claimed to be ‘net zero’ in operation, and required to ‘fuel the transition’ (see, e.g., Offshore Energy UK [36]); airport expansion, in which airlines and airports claim that aviation demand should not be restricted, because emissions can be reduced elsewhere in the economy, and/or technological alternatives to fossil-fuelled aviation will soon be available, and/or flights can be ‘offset’ (see, e,g., IATA [37]); the use of hydrogen for home heating, in which gas companies aggressively promote hydrogen-based solutions for home heating, and associated policies (such as the blending of hydrogen and methane; mandating ‘hydrogen ready’ boilers) despite a strong expert consensus that hydrogen is not best suited to home heating, and should be used for different applications such as industrial uses, with electric heat pumps offering a better alternative [38]; and reliance on GHG removals as ‘offsets’ to compensate for GHG emissions which could have been avoided through other means (see section The role of GHG removals above).

In each of these cases, the evidence points strongly to one conclusion. Yet in each, a false balance is promulgated, ensuring a lively debate in media and policy circles and through legal battles, as happened with the Cumbria mine. Some involved in such debates will be acting in good faith, trying to grapple with a confusing picture. Others will be purposefully introducing complex and conflicting evidence and argumentation, in order to further commercial aims. Whatever the motivation, the overall situation created is one of confusion and uncertainty, slowing the speed of the transition to net zero, creating lengthy legal battles, and putting climate targets in jeopardy.

There are two ways in which these situations could be avoided. First, UK climate legislation could be changed to remove ambiguity and complexity. Second, greater weight could be placed on the quality of evidence used in decision-making. These are discussed in turn below.

### Removing ambiguities in climate legislation

As described above (see section UK climate governance: the state of play) UK climate legislation contains many ambiguities. While the Climate Change Act sets an admirably clear trajectory for GHG emissions over time, the targets and carbon budgets are economy-wide, with little clarity on the relative responsibilities of different government departments, sectors of the economy, or balance between GHG reductions and GHG removals. The following changes would contribute:

- Setting a net zero ‘test’ for all major developments – this was a recommendation in the recent independent Skidmore Review [39].
- Legislation to prevent the opening of new fossil fuel extraction sites, following the example of Wales, who have stated they will not issue permits for new coal mines [20] and in line with the recommendations of the Environmental Audit Committee [40].
- Specific climate targets, responsibilities and powers for local areas on climate change, as recommended by the Climate Change Committee, Skidmore Review and many independent commentators [41].
- Clear responsibilities on climate, linked directly to the CCA budget-setting process, for all government departments and agencies, as recommended by the Climate Change Committee [13].
- A review of the NPPF, to make clear the links between the NPPF and the Climate Change Act, and to specify how all classes of GHG emissions (see section Accounting for GHG emissions) should be taken into account when making planning decisions.
- Separate targets for GHG reductions and removals, enshrined in the CCA budget-setting process [22].

### The quality of evidence used in decision-making

The problem of false balance could be lessened through greater attention being placed on the quality of evidence used in decision-making. There are already-established markers of evidential quality. These include academic peer-review, and publication in quality academic journals; judgements of the standing, independence and expertise of individual specialists; and evidence produced by reputable national and international bodies, such as publicly-funded agencies, international organisations such as international organisations, such as the European Union’s Copernicus Climate Change Service (C3S), the United Nations Environment Programme, the World Meteorological Organization or the Intergovernmental Panel on Climate Change. These are not failsafe indicators of quality. Problems with academic peer-review are well-rehearsed; publicly-funded agencies differ in their independence from government or political groupings; some experts with high standing are wrong. Notwithstanding these problems, the quality of the evidence presented should be a material consideration in decision-making processes. For example, in the Public Inquiry on Woodhouse Colliery, an array of credible experts on climate change, presenting evidence from peer-reviewed or independent sources, should not have been dismissed in favour of the accounts given by the mining company and its consultants who were not climate specialists.

I am not arguing that high-quality ‘expert’ evidence should not be the only type of evidence used or valued in decision-making. For example, it is a longstanding principle that local communities should have a say in decisions that affect them, and there should be no expectation that these representations are peer-reviewed or meet similar evidential standards. However, representations which claim technical or evidential rigour should show transparently how they meet such standards.

A further issue to take into account is the independence of witnesses and evidence provided to policymakers and legal processes such as the Public Inquiry. This is not to say that paid consultants, authoring reports and/or appearing as expert witnesses, are automatically less reliable or less independent. Consultancy can be a useful and necessary way of supplementing in-house expertise. However, there should be greater transparency about financial links and other interests. At the very least, such links should be declared routinely, and taken into account in decision-making. In planning decisions, this would apply both to developers and to other interested parties, such as groups opposing the decision.

There is also a need for organisations making planning decisions, including local authorities and the Planning Inspectorate, to have in-house expertise on climate issues. This would allow them to consider and assess competing claims. The Climate Change Committee has called for guidance for local authorities, on this point [42].

Reducing the ambiguities in current climate legislation and paying closer attention to the quality of evidence used in climate decision-making, would result in quicker and more predictable decisions, and less recourse to lengthy legal battles. This is essential, given the rapid GHG reduction required to meet the net zero goal, and to provide businesses with the certainty and predictability that they require in order to invest in that transition.

## Data Availability

Data sharing not applicable to this article as no datasets were generated or analysed during the current study.
